# Blinatumomab retreatment after relapse in patients with relapsed/refractory B-precursor acute lymphoblastic leukemia

**DOI:** 10.1038/leu.2017.306

**Published:** 2017-10-31

**Authors:** M S Topp, M Stelljes, G Zugmaier, P Barnette, L T Heffner, T Trippett, J Duell, R C Bargou, C Holland, J E Benjamin, M Klinger, M R Litzow

**Affiliations:** 1 Medizinische Klinik und Poliklinik II, Universitätsklinikum Würzburg, Würzburg, Germany; 2 Hematology, Oncology and Pneumology, University of Münster, Münster, Germany; 3 Research and Development, Amgen Research (Munich) GmbH, Munich, Germany; 4 Pediatric Hematology/Oncology, Primary Children's Hospital, Salt Lake City, UT, USA; 5 Winship Cancer Institute, Emory University School of Medicine, Atlanta, GA, USA; 6 Memorial Sloan-Kettering Cancer Center, New York, NY, USA; 7 Comprehensive Cancer Center Mainfranken, University Hospital, Würzburg, Germany; 8 Research and Development, Amgen Inc., Washington, DC, USA; 9 Research and Development, Amgen Inc., Thousand Oaks, CA, USA; 10 Division of Hematology, Mayo Clinic, Rochester, MN, USA

Relapsed/refractory B-precursor acute lymphoblastic leukemia (ALL) is an aggressive malignant disease with poor prognosis and an unmet medical need. The prognosis is particularly dismal for adults with ALL who require second salvage therapy or children who require multiple salvage therapies after prior therapies have failed.^1, 2^ Blinatumomab is a Bispecific T-cell Engager (BiTE) antibody construct that is designed to link T cells and CD19-positive B cells, inducing tumor cell lysis. Three single-arm, open-label phase 2 studies in patients with relapsed/refractory ALL reported antileukemia activity for blinatumomab, including one study of pediatric ALL^3^ and two studies of adult ALL.^4, 5^ In a randomized, open-label, phase 3 study, overall survival improved with blinatumomab compared with standard of care chemotherapy in adults with relapsed/refractory ALL.^6^ In phase 2 and phase 3 studies in adults,^5, 6^ remission rates did not significantly differ by the number of prior lines of therapy.

In each of the three single-arm, open-label, phase 2 studies, blinatumomab retreatment was permitted if a patient relapsed after an initial response to blinatumomab. The objective of this analysis was to evaluate the efficacy of blinatumomab retreatment in these patients. Study MT103-205 (NCT01471782; EudraCT 2010-024264-18) was a phase 1–2 study of 93 children and adolescents with B-precursor ALL that was in second or later bone marrow relapse (blast percentage >25%), in relapse after allogeneic hematopoietic stem cell transplantation (alloHSCT), or refractory to other treatments.^3^ Study MT103-206 (NCT01209286; EudraCT 2009-015989-62) was an exploratory phase 2 study of 36 adult patients with B-precursor relapsed/refractory ALL and >5% marrow blasts.^4^ Study MT103-211 (NCT01466179; EudraCT 2011-002257-61) was a confirmatory phase 2 study of 189 adult patients with Philadelphia chromosome-negative B-precursor ALL that was primary refractory, in early (⩽12 months) first relapse, in early relapse after alloHSCT, or in later relapse; patients with ⩾10% marrow blasts were eligible. Key exclusion criteria in each study were alloHSCT in the previous 3 months, active graft-versus-host disease, or central nervous system involvement.

Each cycle consisted of 4 weeks of blinatumomab by continuous infusion, followed by a 2-week treatment-free interval. Studies MT103-205 and MT103-206 included a dose-finding part and the selected dose was defined by body surface area, with stepwise dosing of 5–15 μg/m^2^/day (5 μg/m^2^/day in cycle 1 week 1 and 15 μg/m^2^/day thereafter). In Study MT103-211, all patients received stepwise fixed dosing of 9–28 μg/day (9 μg/day in cycle 1 week 1 and 28 μg/day thereafter). After each infusion period, bone marrow aspiration was performed to evaluate efficacy. Lumbar puncture was performed after bone marrow aspiration to evaluate central nervous system leukemic involvement and for the administration of intrathecal chemotherapy prior to start of blinatumomab and after each cycle.

Response criteria for each study are provided in Supplementary Table S1. Patients who achieved a remission within the first two cycles could receive up to three additional cycles of blinatumomab or alloHSCT instead of further blinatumomab treatment. If the patient experienced hematologic relapse during follow-up, up to three additional cycles of blinatumomab retreatment could be administered. For patients from Study MT103-205, retreatment was stepwise dosing of 5–15 μg/m^2^/day. For patients from Studies MT103-206 and MT103-211, retreatment was administered with the patient’s original dosing schedule. Adverse events were recorded throughout initial treatment and retreatment. An Independent Ethics Committee or Institutional Review Board responsible for each site approved each of the study designs. Informed consent was obtained from all patients.

Eleven patients (seven male, four female) received blinatumomab retreatment after initial response and relapse. The median age of retreated patients was 25 years (range, 4–77); two were <18 years, eight were 18 to <65 years, and one was ⩾65 years. Before original study enrollment, these patients had experienced one (*n*=5), two (*n*=4), or three or more (*n*=2) prior relapses. Bone marrow blast infiltration before blinatumomab treatment was ⩾50% for seven patients and <50% for four patients. The median duration of initial blinatumomab treatment for these 11 patients was 82 days (range, 1–150). The median duration of initial blinatumomab-induced response was 9.3 months (range, 3.5–12.4), and the median duration of the treatment-free period between initial treatment and retreatment with blinatumomab was 6.6 months (range, 3.0–12.2). Between initial treatment and retreatment, nine patients were CD19-positive by flow cytometry, one was CD19-positive by immunocytochemistry, and one had unknown CD19 status. The median duration of blinatumomab retreatment was 28 days (range, 4–85).

Clinical characteristics for individual patients are summarized in Table 1. Seven patients had received alloHSCT before initial blinatumomab treatment and six had received alloHSCT between blinatumomab treatment and retreatment. Between blinatumomab treatment and retreatment, one patient received chemotherapy (FLAG-IDA) followed by alloHSCT. One patient received blinatumomab retreatment twice. Three patients received additional systemic therapy after blinatumomab retreatment.

Four patients (36%) responded to retreatment, all in the first cycle. All four responders were adults and were CD19-positive: three by flow cytometry and one by immunocytochemistry. Median overall survival for all retreated patients was 9.4 months (95% confidence interval: 0.7, 12.9) from the start of blinatumomab retreatment (Supplementary Figure S1). Among the four responders to blinatumomab retreatment, overall survival after initiation of blinatumomab retreatment was 4.8 months for one patient and overall survival was censored at 3.7, 4.6, and 20.0 months, respectively, in the other three patients who were in ongoing survival at the time of data cutoff. Per the study designs, each of these patients achieved remission with blinatumomab retreatment as second or later salvage therapy for ALL.

Adverse events with blinatumomab retreatment (Table 2) were consistent with those reported with initial blinatumomab treatment.^3, 4, 5^ Three patients (27%) had grade ⩾3 neurologic events during retreatment; all three patients also had neurologic events during initial blinatumomab treatment. A 77-year-old man in Study MT103-206 had grade 3 treatment-related encephalopathy with initial treatment that recurred with retreatment. Blinatumomab retreatment was discontinued and the event resolved. The patient achieved a partial remission with blinatumomab retreatment. A 21-year-old man in Study MT103-206 had grade 1 neurologic events in initial treatment that were not related to blinatumomab; grade 3 treatment-related epilepsy during blinatumomab retreatment resolved with permanent discontinuation of blinatumomab. The patient’s response to retreatment was not assessed. A 20-year-old woman in Study MT103-211 had grade 2 treatment-related disorientation with initial treatment and grade 3 paraplegia in retreatment that was not related and resolved with continued blinatumomab retreatment. The patient achieved a complete remission with blinatumomab retreatment. There were no reports of cytokine release syndrome with blinatumomab retreatment.

T-cell and B-cell kinetics during initial blinatumomab treatment and during blinatumomab retreatment were recorded for the five patients in Study MT103-206 (Supplementary Figure S2). In these patients, all of whom had complete remission, partial remission, or a hypocellular response to blinatumomab retreatment, some T-cell expansion and sustained B-cell depletion were also observed with blinatumomab retreatment.

Blast counts at the relapse that led to blinatumomab retreatment were recorded for four patients (Table 1): 10% before complete remission, 10% before partial remission, 10% before response not assessed but overall survival of 12.3 months, and 93% before no response to retreatment. The lack of response in the patient with 93% blast count is consistent with published demonstration of higher remission rates with initial blinatumomab treatment in patients with counts below 50% than in patients with counts of at least 50%, including both pediatric patients (56 versus 33%)^3^ and adult patients (73 versus 29%).^5^ As described above, grade ⩾3 neurologic adverse events were observed with blinatumomab retreatment in three patients: two with related events and one with an unrelated event. The two patients with related neurologic events survived for more than 12 months. But information about blast counts and neurotoxicity in individual patients (Table 1) was not sufficiently informative and the sample size in this analysis was too small for definitive conclusions about patient-specific factors that predict response to blinatumomab retreatment. However, these results suggest that T-cell and B-cell kinetics, blast counts, and adverse events could be analyzed as possible predictors in a larger study of blinatumomab retreatment. Other patient-specific factors such as CD19 expression or expression of the programmed death receptor and its ligand (PD-1/PD-L1)^7, 8^ were not recorded in these studies and could also be considered for future research.

In conclusion, this analysis showed that blinatumomab retreatment may be successful in patients with relapsed/refractory ALL and prior responses to blinatumomab, even among those who have an early CD19-positive relapse within 12 months. Despite the small sample size, the remission rate for blinatumomab retreatment in this analysis (36%) was similar to that recently reported for initial blinatumomab treatment in a large, randomized, phase 3 pivotal study (44%).^6^ These results suggest that blinatumomab retreatment is a reasonable intervention for relapse among patients who have responded to blinatumomab previously. Additional study of a larger population would be required to identify predictors of response to blinatumomab retreatment.

## Figures and Tables

**Table 1:** Relapse-free survival and overall survival after blinatumomab retreatment

*Pt*	*Age*^a^	*Study*	*Initial treatment*			*Intervening alloHSCT*	*Retreatment*			*Subsequent systemic therapy*	*RFS (months)*	*Overall survival (months)*
			*Daily dose*	*Response duration (months)*	*Blasts at relapse*		*Daily dose*	*Grade* ⩾*3 neurologic event*	*Best hematologic response*			
1	12	205	15–30 μg/m^2^	12.4	—		5–15 μg/m^2^		PD			0.7^b^
2	4		5–15 μg/m^2^	3.4	93%	Yes	5–15 μg/m^2^		No response	Yes		6.7
3	77	206	15 μg/m^2^	7.8	10%		15 μg/m^2^	Yes	PR			12.9
4^c^	38		5–15–30 μg/m^2^	17.5	10%		5–15–30 μg/m^2^		CR		8.6	
					32%		5–15–30 μg/m^2^		CR	Yes	9.7	20.0^b^
5	24		5–15 μg/m^2^	7.6	—	Yes	5–15 μg/m^2^		Hypocellular			9.4
6	62		15 μg/m^2^	d	—	Yes	15 μg/m^2^		CRh		3.9	4.8
7	21		5–15 μg/m^2^	10.6	10%		5–15 μg/m^2^	Yes	—			12.3
8	20	211	9–28 μg	14.2	—		9–28 μg	Yes	CR	Yes	1.7	3.7^b^
9	29		9–28 μg	10.5	—	Yes	9–28 μg		PD			0.7
10	26		9–28 μg	5.1	—	Yes	9–28 μg		PD			1.8
11	25		9–28 μg	11.3	—	Yes	9–28 μg		CRh		3.7	4.6^b^
										Median (95% CI)	3.8 (1.7, 8.6)	9.4 (0.7, 12.9)

Abbreviations: —, not assessed; alloHSCT, allogeneic hematopoietic stem cell transplantation; CI, confidence interval; CR, complete remission; CRh, CR with partial hematologic recovery; PD, progressive disease; PR, partial remission; RFS, relapse-free survival.

a Age, in years, before initial treatment.

b Censored observation (ongoing survival).

c This patient received blinatumomab retreatment twice.

d Duration of response was not calculated for this patient because the initial response occurred after blinatumomab treatment ended.

**Table 2:** Adverse events in >1 retreated patient overall, by age group

	*Incidence in retreatment*, n *(%)*		
	*Adult (*n*=9)*	*Children and Adolescents (*n*=2)*	*All (*N*=11)*
Any adverse event	9 (100)	1 (50)	10 (91)
Pyrexia	7 (78)	1 (50)	8 (73)
Diarrhea	3 (33)	1 (50)	4 (36)
Leukopenia	3 (33)	0 (0)	3 (27)
Neutropenia	3 (33)	0 (0)	3 (27)
Thrombocytopenia	3 (33)	0 (0)	3 (27)
Anemia	2 (22)	0 (0)	2 (18)
Cough	2 (22)	0 (0)	2 (18)
Epistaxis	1 (11)	1 (50)	2 (18)
Fatigue	2 (22)	0 (0)	2 (18)
Febrile neutropenia	1 (11)	1 (50)	2 (18)
Muscular weakness	2 (22)	0 (0)	2 (18)
Myopathy	2 (22)	0 (0)	2 (18)
Edema	2 (22)	0 (0)	2 (18)
Paresthesia	2 (22)	0 (0)	2 (18)
Tachycardia	2 (22)	0 (0)	2 (18)
Weight decreased	2 (22)	0 (0)	2 (18)
Weight increased	2 (22)	0 (0)	2 (18)
Any adverse event grade ⩾3	7 (78)	1 (50)	8 (73)
Neutropenia	3 (33)	0 (0)	3 (27)
Thrombocytopenia	3 (33)	0 (0)	3 (27)
Anemia	2 (22)	0 (0)	2 (18)
Febrile neutropenia	1 (11)	1 (50)	2 (18)
