# Supplementary material for: Blinatumomab retreatment after relapse in patients with relapsed/refractory B-precursor acute lymphoblastic leukemia
Source: Leukemia. 2017 Oct 31;32(2):562–5. doi: 10.1038/leu.2017.306 (PMC5808068; doi:10.1038/leu.2017.306)
Supplement: Supplementary Information [file leu2017306x1.docx]

# Supplementary Data

## Supplementary Table S1. Response criteria for each study

| Response | Study MT103-205 | Study MT103-206 | Study MT103-211 |
| --- | --- | --- | --- |
| Complete remission (CR) | No evidence of circulating blasts or extramedullary disease: <5% blasts in bone marrow (M1 bone marrow)  Subclassifications:   - full recovery of peripheral blood counts: platelets >100,000/µl and ANC >1000/µl - incomplete recovery of peripheral blood counts: platelets >50,000/µl but ≤100,000/µl; ANC >500/µl but ≤1000/µl | ≤5% blasts in bone marrow  No evidence of disease  Full recovery of peripheral blood counts: platelets >100,000/μl, ANC >1500/μl hemoglobin ≥11 g/dl | ≤5% blasts in bone marrow  No evidence of disease  Full recovery of peripheral blood counts: platelets >100,000/μl and ANC >1000/μl |
| Complete remission with partial hematologic recovery (CRh) | - | ≤5% blasts in bone marrow  No evidence of disease  Full recovery of peripheral blood counts: platelets >50,000/μl, ANC >500/μl, and hemoglobin ≥7 g/dl | ≤5% blasts in bone marrow  No evidence of disease  Partial recovery of peripheral blood counts: platelets >50,000/μl and ANC >500/μl |
| Blast-free hypoplastic or aplastic bone marrow | - | - | ≤5% blasts in bone marrow  No evidence of disease |
| Partial remission | Complete disappearance of circulating blasts and achievement of M2 marrow status (≥5% or <25% blast cells) and appearance of normal progenitor cells | ≤25% blasts in bone marrow | 6%–25% blasts in bone marrow with a ≥50% reduction from baseline |
| Hematological relapse | >25% blasts in bone marrow after documented CR  An extramedullary relapse was considered a relapse and was subdivided into CD19-positive and CD19-negative | >5% blasts in bone marrow after documented CR/CRh  An extramedullary relapse was assessed as hematological relapse | >5% blasts in bone marrow, or blasts in peripheral blood after documented CR/CRh  An extramedullary relapse was assessed as hematological relapse |

Abbreviations: ANC, absolute neutrophil count.

**Supplementary Figure S1.** Overall survival from the start of blinatumomab retreatment.

The plot does not include overall survival between initial blinatumomab treatment and blinatumomab retreatment; the response duration for the initial response to blinatumomab is provided for each patient in Table 1.


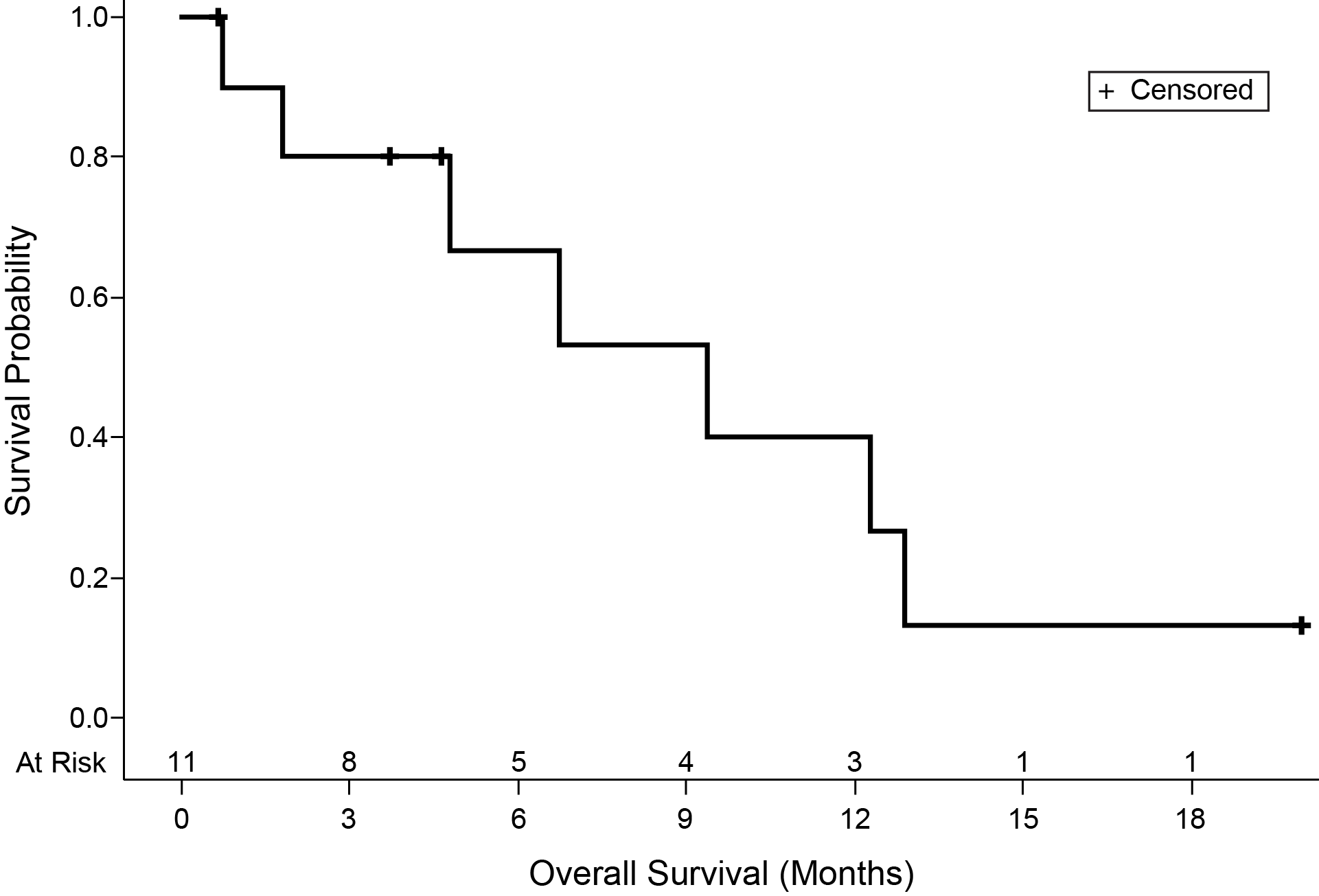


**Supplementary Figure S2.** T-cell and B-cell kinetics for individual patients at weekly assessments
